# Topical Volumizing Cream Improves Facial Volume and Skin Health in Adults With Rapid Weight Loss From Pharmacologic (GLP‐1/GIP Agonists), Surgical, or Behavioral Interventions

**DOI:** 10.1111/jocd.70681

**Published:** 2026-01-20

**Authors:** Nhi Nguyen, Alejandra Aguilar, Nasima Afzal, Andy Lee, Wardah Akram, Minh N. Duong, Raja K. Sivamani

**Affiliations:** ^1^ Integrative Research Institute Sacramento California USA; ^2^ California Northstate University, College of Medicine Elk Grove California USA; ^3^ Pacific Skin Institute Sacramento California USA

**Keywords:** elasticity, facial aging, GIP, GLP‐1, volume, weight loss, wrinkles

## Abstract

**Background:**

GLP‐1 receptor agonists (GLP‐1) and dual GLP‐1/gastric inhibitory polypeptide (GIP) agonists emerged in recent years to promote weight loss. By reducing subcutaneous fat, collagen, and overall structural support of the skin, rapid weight loss can lead to visual face aging.

**Aims:**

This study investigates the synergistic effect of a multi‐ingredient topical that acts on different skin levels to treat facial aging in individuals experiencing rapid weight loss from GLP‐1 and GIP/GLP‐1 agonist use.

**Patients/Methods:**

In this 12‐week study, 33 participants from the Sacramento region were given the same topical volumizing cream (Vol.U.Lift, Image Skincare). Participants had 6 visits: screening, baseline, weeks 2, 4, 8, and 12. Tolerability assessments, facial photography, and measurements of hydration, elasticity, and wrinkles were acquired.

**Results:**

29 subjects completed all study related procedures. Wrinkle severity appearance decreased at week 4 (14.5%, *p* < 0.001) and 12 (20.7%, *p* < 0.001). The ultrasound based skin and subcutaneous thickness increased at week 4 (17.3%, *p* = 0.04) and 12 (20.1%, *p* = 0.015); TEWL decreased at week 4 (10.39%, *p* ≤ 0.02) and 12 (12.7%, *p* ≤ 0.02); Skin hydration increased at week 2 (21.29%, *p* ≤ 0.01), 4 (19.50%, *p* ≤ 0.02), and week 12 (21.8%, *p* ≤ 0.02); Firmness (22.5%, *p* ≤ 0.002), viscoelasticity (13.1%, *p* ≤ 0.02) and net elasticity (23.2%, *p* ≤ 0.0002) increased at week 12.

**Conclusions:**

The use of a topical volumizing cream improved the appearance of facial volume, wrinkles, and biophysical characteristics in participants who had undergone rapid weight loss primarily due to GLP‐1/GIP agonist use.

## 1 Introduction

GLP‐1 receptor agonists (GLP‐1) and dual GLP‐1/Gastric inhibitory polypeptide (GLP‐1/GIP) agonists have emerged in recent years to promote weight loss [1]. These medications promote the secretion of insulin, inhibit glucagon production from pancreatic α‐cells, and decrease pancreatic β‐cell apoptosis [2]. This mechanism of action allows suppression of appetite, delay in gastric emptying, and reduction in calorie intake, with FDA approvals for the treatment of conditions such as type 2 diabetes and obesity. Furthermore, enhanced GLP‐1 secretion in patients with bariatric surgery is thought to counteract the antagonistic effect of exendin‐9 that decreases insulin secretion [3]. The weight loss that can occur secondary to a GLP‐1/GIP agonist can be observed as early as 8–12 weeks [4, 5]. However, there is a growing recognition that significant weight loss may accelerate the visual signs of facial aging such as the decrease in adipose tissue volume, skin elasticity, and the thickness and density of collagen fibers [6, 7, 8].

Although numerous studies have investigated the promising benefits of rapid weight loss induced by GLP‐1 receptor agonists [4, 9, 10, 11] there is a lack of research examining the implications of such rapid weight loss on skin aging. Notably, while many products are available to individually target aspects of skin aging [12, 13, 14, 15, 16], there is a lack of topical formulations that combine proven ingredients to specifically address the accelerated skin aging associated with GLP‐1–induced weight loss.

Significant weight loss, such as from the use of GLP‐1/GIP agonists, is associated with both loss of muscle and connective tissue along with the loss of fat [17]. In turn, there is an associated loss of facial volume as a result of fat, muscle, and connective tissue loss that has been termed “Ozempic Face” [18]. Collagen turnover, determined by the equilibrium between collagen production and breakdown, occurs between 80 and 120 days [19]. Studies suggest that GLP‐1 acts on the receptor of adipose‐derived stem cells (ADSCs) [20], thereby inhibiting collagen synthesis and increasing the activity of matrix metalloproteinases (MMP‐1), resulting in a net loss of elastic and collagen [21, 22, 23]. Overall, the sudden loss of facial volume can accelerate the visible signs of aging. While there are many traditional topical therapies that are available for skin aging, such as the retinoids and antioxidants, they typically do not lead to volume restoration of face beyond the epidermis and the superficial dermis.

For example, superficial bakuchiol, hyaluronic acid (HA), and antioxidants have been shown to reduce the appearance of wrinkles, lead to more even skin tone, and boost resistance to oxidative stress [12, 13, 15, 16, 24]. While retinoids are well known for their ability to modulate skin aging superficially [25] their tolerability has led to alternative ingredients such as bakuchiol to be in demand [16, 24]. With its relatively higher tolerability and retinol‐like properties to modulate genes that regulate extracellular matrix production and dermal‐epidermal junction, it enhances human fibroblast cell activity and inhibits the expression of MMP‐1 [24, 26]. Antioxidants such as vitamin C and astaxanthin, especially when used in tandem, are also highly sought after due to its ability to prevent the acceleration of skin damage from reactive oxygen species (ROS) by neutralization [27, 28]. Vitamin C is a potent antioxidant that is most commonly found in the form of L‐ascorbic acid or Tetrahexyldecyl ascorbate and provides protection against environmental and UV‐induced ROS. In addition to activating transcription factors that aid in the synthesis and stabilization of collagen, it prevents collagen degradation by promoting the synthesis of the MMP‐1 tissue inhibitor [29, 30]. Similarly, astaxanthin exhibits antioxidant comparable capabilities in mitigating oxidative stress. By scavenging singlet oxygen produced by UV rays in the epidermis and dermis, astaxanthin prevents the UV‐induced skin deterioration. HA is well known for its anti‐aging effects and humectant properties, which enhance skin hydration and volume [28, 31]. At specific molecular weights, HA can penetrate the stratum corneum, modulate epidermal cell interactions, and increase the skin's water‐holding capacity [28, 32].

Several ingredients work on cell types that are found deeper in the skin as well. Extracts of *Anigozanthos flavidus* (kangaroo paw) have been shown to improve fibroblast production of tenascin‐X, collagen, and elastin production in fibroblasts, and a clinical study with its use topically showed improvement in signs of facial aging and skin elasticity [33]. The amino acid L‐ornithine has been shown to reduce lipolysis in adipocytes, thereby leading to more retention of fat in the adipocytes (patent WO2015167557A1, Publication Date: 2015‐11‐05, Application Number: US2014/036233, Filing Date: 2014‐04‐30). Although both ingredients have cell culture support for addressing deeper layers in the skin, there have been few published clinical studies.

The aim of this clinical trial is to investigate the synergistic effect of a topical cream containing multiple ingredients that are known to work on different cell types in various levels of the skin in individuals experiencing rapid weight loss due to use of GLP‐1 and GIP/GLP‐1 agonists. The goal of the study was to assess whether topical use can counteract the visible loss of volume and the signs of aging.

## 2 Materials and Methods

### 2.1 Subjects

This 12‐week prospective, open‐label clinical study was conducted in the Sacramento region at Integrative Research Institute from November 2024 to June 2025. Inclusion criteria included healthy men and women between the ages of 35 and 65 years old experiencing skin changes from a history of self‐reported rapid weight loss within the last 1 year (defined as equal to or greater than 10% of total body weight) due to one of the following: GLP‐1 agonist, GIP/GLP‐1 receptor co‐agonists, alternative weight loss promoting drugs, gastric bypass surgery, or other weight loss promoting behavior. Individuals must also experience at least two skin changes including sagging skin or jawline, loss of volume, loss of firmness, prominent nasolabial folds, marionettes, wrinkles under the eye area, loose skin, dullness or lack of radiance, ashy skin, uneven skin, or blotchiness. Exclusion criteria included pregnant or breastfeeding people, individuals who modified their hormonal‐based contraception within 3 months prior to study entry, current tobacco smokers, individuals with an excess of a 10 pack‐year history of tobacco use, prisoners, or subjects who were otherwise unable to provide consent, individuals with insulin‐controlled Type 1 diabetes mellitus or who had an autoimmune photosensitive condition, a known genetic condition associated with decreased collagen production, a skin disease that would affect assessments per investigator, or any known allergy to any of the ingredients in the study product were also excluded. Individuals with a history of malignancy besides localized skin cancer within the past year, on chemotherapy or immunotherapy, or use redlight therapy within 1 month of joining the study or were unwilling to refrain from it during the duration of the study were excluded. Individuals who had undergone facial cosmetic procedures or treatments within the past 6 months or those who were unwilling to refrain from such procedures during the duration of the study were excluded. Additionally, individuals who had used topical antioxidant ingredients such as vitamin C or vitamin E, topical pigment reducing agents such as hydroquinone, azelaic acid, tranexamic acid, kojic acid, topical retinoids, bakuchiol, or any topical vitamin A derivative within 2 weeks before enrollment or those unwilling to undergo a washout period prior to enrollment were also excluded from participation. Individuals were also instructed to undergo a four‐week washout on oral collagen supplementation. This study protocol was approved by the Allendale Institutional Review Board (Protocol # IRI24_08_ImageSkin_OTIS on November 14, 2024) and all subjects provided signed informed consent prior to participation.

### 2.2 Investigational Products

All participants were allocated to receive the study intervention (Vol.U.Lift from Image Skincare) in unmarked tubes along with a commercially available sunscreen (SPF 75). Participants were instructed to apply the study product twice daily to clean skin in the morning and evening, including the neck and jawline. Participants were also given a product log to record daily usage as instructed until their follow‐up visit.

### 2.3 Study Visits and Procedures

This study comprised a total of six in‐person clinic visits, which included: screening and consent, baseline assessment, and follow‐up visits at weeks 2, 4, 8, and 12. TEWL, skin hydration, and skin elasticity and firmness were assessed using the VapoMeter (Delfin Technologies, Kuopio, Finland), Skin MoistureMeterSC (Delfin Technologies, Kuopio, Finland), and Cutomer MPA 580 (Courage+Khazaka electronic GmbH, Germany), respectively, at the baseline and follow‐up visits. Facial photographs were captured through the VISIA PRIMOS (Canfield, Parsippany, NJ). At baseline, week 4, and week 8, an ultrasound device (Vscan Air CL, GE Healthcare, Chicago, IL) was used to measure the thickness of the skin from the epidermis to the subcutis. A clinical grading of facial wrinkles was also conducted at each visit starting from baseline.

### 2.4 Statistical Analysis

The Shapiro–Wilk test was utilized to assess normality, and baseline comparisons were performed using paired, two‐tailed Student's *t*‐tests. Statistical significance was set at *p* ≤ 0.05. Results were presented as mean with standard error of mean. Data were compared within each group against baseline and then between the two treatment groups at each time point. For data visualization, Prism v. 10 (GraphPad Software LLC, San Diego, CA, USA), a statistical graphing software, was used. The data was analyzed as observed when accounting for dropouts.

## 3 Results

**FIGURE 1:**
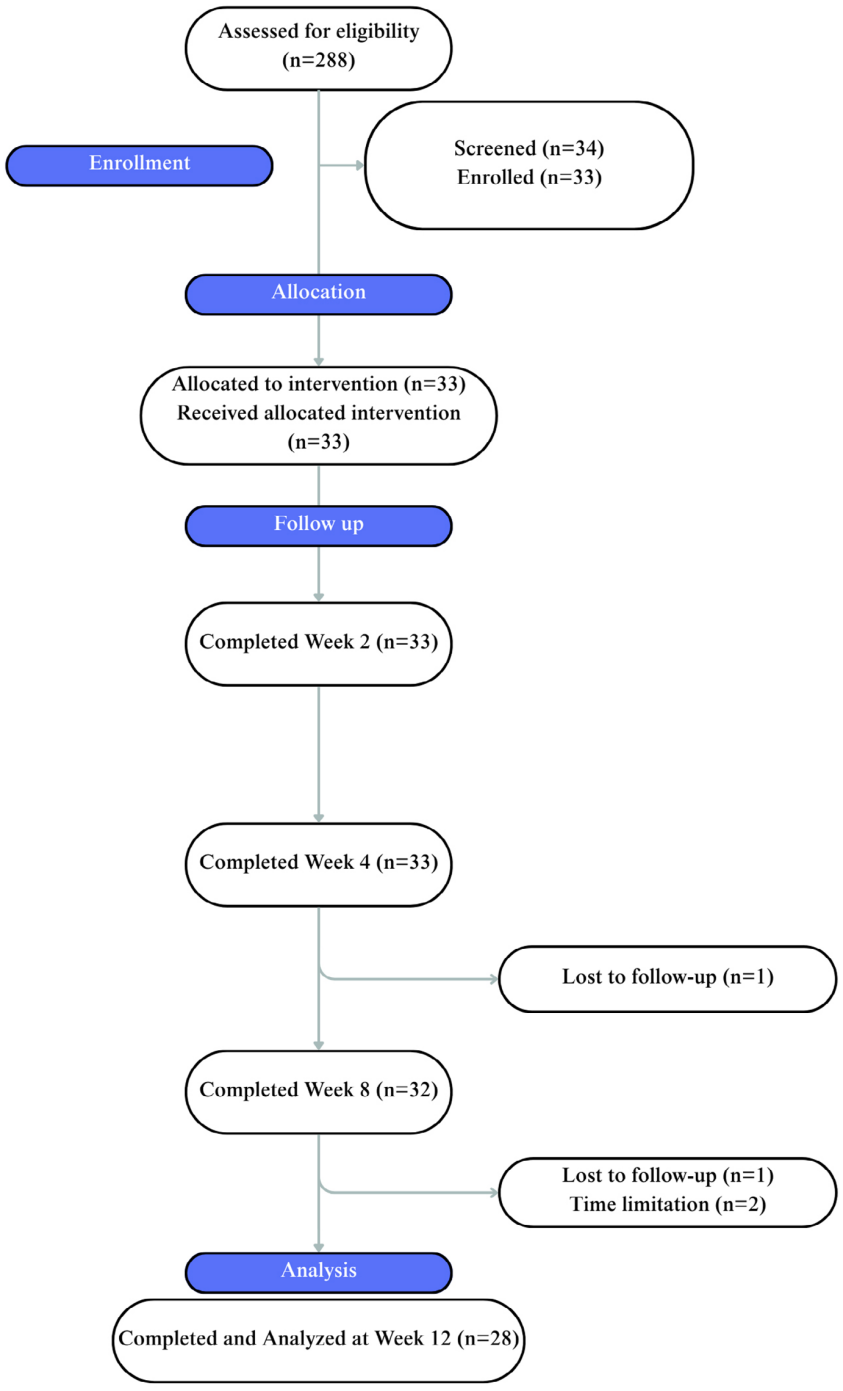
CONSORT diagram for clinical study.

**FIGURE 2:**
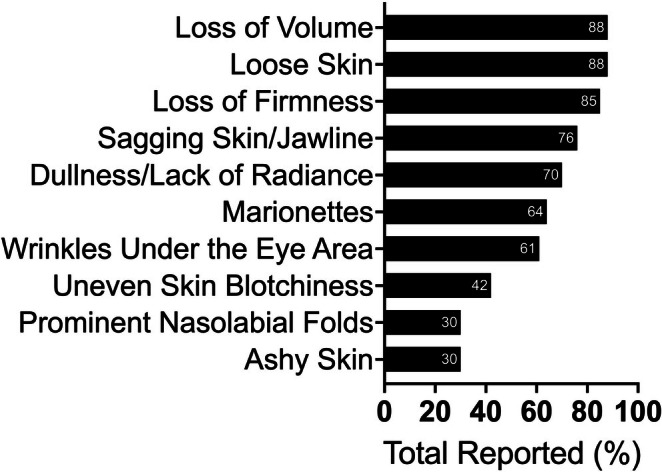
Subjective reports of skin changes exacerbated due to the rapid weight loss.

A total of 288 people were assessed for eligibility and 33 participants were enrolled (Figure 1). A total of 29 participants completed all visits and were included in the analysis. Two participants were lost to follow‐up, where one was using a GLP‐1 agonist and one had bariatric surgery. From the 2 participants who withdrew due to time limitations, one was on a GLP‐1 agonist and one was on a GIP/GLP‐1 co‐agonist. In total, there were 33 participants enrolled with an average age of 49 ± 8 years. Two subjects were male, and the rest of the subjects were female. The majority of participants were on a GLP‐1 agonist (*n* = 15) or GIP/GLP‐1 co‐agonist (*n* = 9). The rest of the subjects had bariatric surgery (*n* = 5), alternate weight loss medication (*n* = 1), or other weight loss promoting behavior (*n* = 3). This is shown in Tables 1 and 2. The most reported skin changes exacerbated due to the rapid weight loss were loss of volume, loss of firmness, and loose skin (Figure 2).

**TABLE 1:** Biologic medication induced weight loss demographics.

	Type	Duration
GLP‐1 agonist (*n* = 15)	Semaglutide	5 months
	Semaglutide	11 months
	Semaglutide	15 months
	Semaglutide	24 months
	Semaglutide	35 months
	Semaglutide	5 months
	Semaglutide	15 months
	Semaglutide	17 months
	Semaglutide	12 months
	Semaglutide	15 months
	Semaglutide	14 months
	Semaglutide	13 months
	Semaglutide	11 months
	Semaglutide	18 months
	Semaglutide	14 months
GIP/GLP‐co‐agonist (*n* = 9)	Tirzepatide	12 months
	Tirzepatide	9 months
	Tirzepatide	12 months
	Tirzepatide	3 months
	Tirzepatide	7 months
	Tirzepatide	12 months
	Tirzepatide	17 months
	Tirzepatide	14 months
	Tirzepatide	6 months

**TABLE 2:** Etiology of non‐biologic medication induced weight loss (other).

Type	Total (*n* = 9)
Gastrectomy sleeve (bariatric) surgery	*n* = 5
Other weight loss medication (topomax)	*n* = 1
Other weight loss promoting behavior (diet)	*n* = 2
Other weight loss promoting behavior (stress)	*n* = 1

### 3.1 Clinical Grading of Wrinkle Severity

**FIGURE 3:**
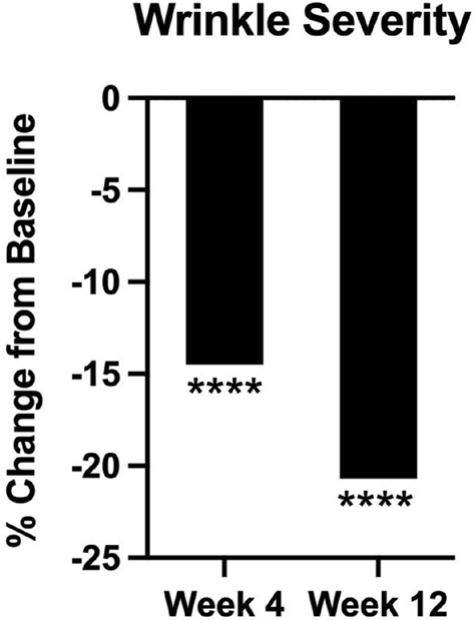
Percent change in clinical grading of wrinkle severity at weeks 4 and 12 compared to baseline. ****p ≤ 0.001.

Clinical grading of wrinkle severity is shown in Figure 3. At weeks 4 and 12, there is a significant reduction in the dermatologist graded appearance of facial wrinkle severity by 14.5% (*p* < 0.001) and 20.7% (*p* < 0.001), respectively.

### 3.2 Ultrasound‐Based Thickness

**FIGURE 4:**
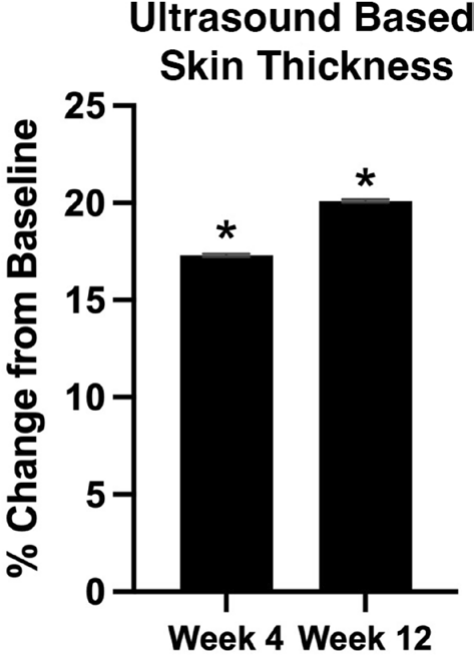
Percent change in ultrasound based facial skin and subcutaneous thickness at week 4 and week 12 compared to baseline. *p ≤ 0.05.

Ultrasound‐based thickness is shown in Figure 4. At weeks 4 and 12, the ultrasound‐based thickness of facial cheek skin and subcutaneous fat increased by 17.3% (*p* = 0.04) and 20.1% (*p* = 0.015), respectively.

### 3.3 Pliability/Firmness

**FIGURE 5:**
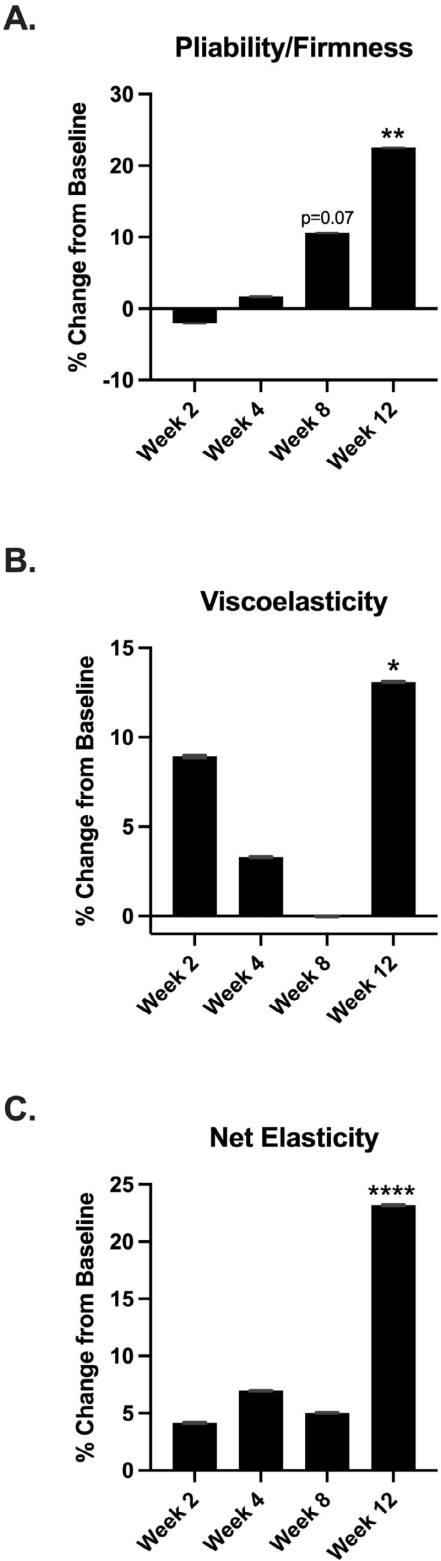
(A) Firmness percent change at week 2, week 4, week 8, and week 12 compared to baseline. (B) Viscoelasticity percent change at week 2, week 4, week 8, and week 12 compared to baseline. (C) Net elasticity percent change at week 2, week 4, week 8, and week 12 compared to baseline. *p ≤ 0.05, **p ≤ 0.01, ****p ≤ 0.001.

Pliability/Firmness is shown in Figure 5A. There was a trending increase of 10.59% at week 8 (*p* = 0.07) and a significant increase of 22.5% at week 12 (*p* ≤ 0.002) when compared to baseline.

### 3.4 Viscoelasticity

Viscoelasticity is shown in Figure 5B. There was a significant increase of 13.1% at week 12 (*p* ≤ 0.02) when compared to baseline.

### 3.5 Net Elasticity

Net Elasticity is shown in Figure 5C. There was a significant increase of 23.2% at week 12 (*p* ≤ 0.0002) when compared to baseline.

### 3.6 Skin Hydration

**FIGURE 6:**
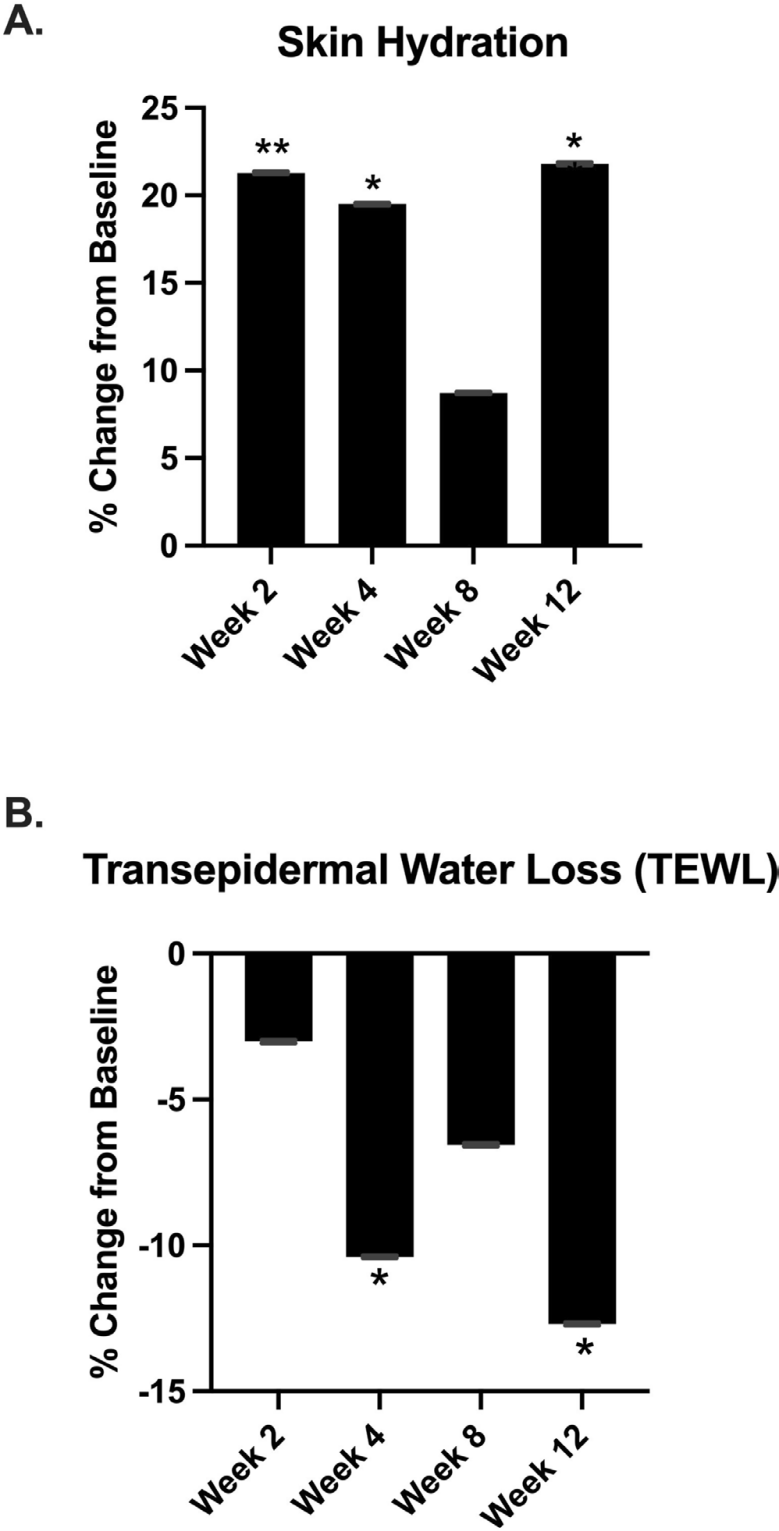
(A) Skin hydration percent change at week 2, week 4, week 8, and week 12 compared to baseline. (B) TEWL percent change at week 2, week 4, week 8, and week 12 compared to baseline. *p ≤ 0.05, **p ≤ 0.01.

Skin hydration is shown in Figure 6A. There was a significant increase of 21.29% at week 2 (*p* ≤ 0.01), 19.50% at week 4 (*p* ≤ 0.02), and 21.80% at week 12 (*p* ≤ 0.02) when compared to baseline.

### 3.7 Transepidermal Water Loss (TEWL)

TEWL is shown in Figure 6B. There was a significant decrease of 10.39% at week 4 (*p* ≤ 0.02) and 12.7% at week 12 (*p* ≤ 0.02) when compared to baseline.

### 3.8 Tolerability

Subjective ratings of tolerability are shown in Table 4. The 3‐point scale referred to “0” as none, “1” as mild, “2” as moderate, and “3” as severe. By week 12, 100% reported none to any itching, burning, stinging, hypopigmentation, or hyperpigmentation. 93% reported none to any redness at week 12.

### 3.9 Photography

**FIGURE 7:**
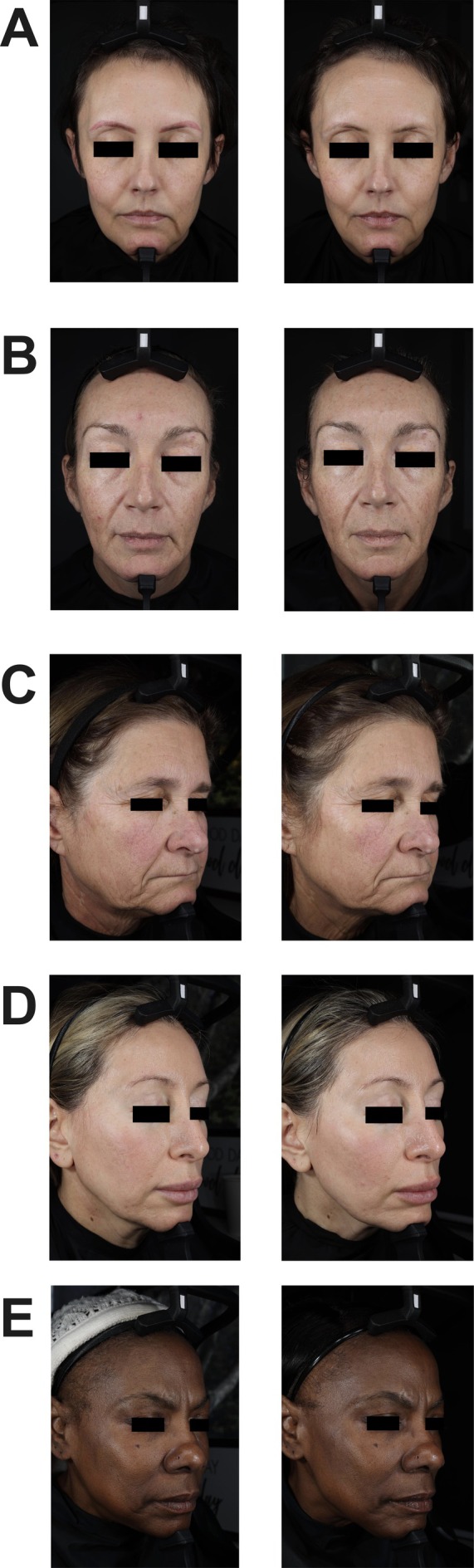
Facial photography of participants. (A) Female, 44 years old, 15 months on a GLP‐1 agonist. Frontal view at baseline and week 12. (B) Female, 47 years old, 17 months on a GLP‐1 agonist. Frontal view at baseline and week 12. (C) Female, 61 years old, 5 months on a GLP‐1 agonist. Right oblique view at baseline and week 4. (D) Female, 46 years old, 9 months on a GIP/GLP‐1 co‐agonist. Right oblique view at baseline and week 12. (E) Female, 60 years old, 12 months on a GLP‐1 agonist. Right oblique view at baseline and week 12.

**FIGURE 8:**
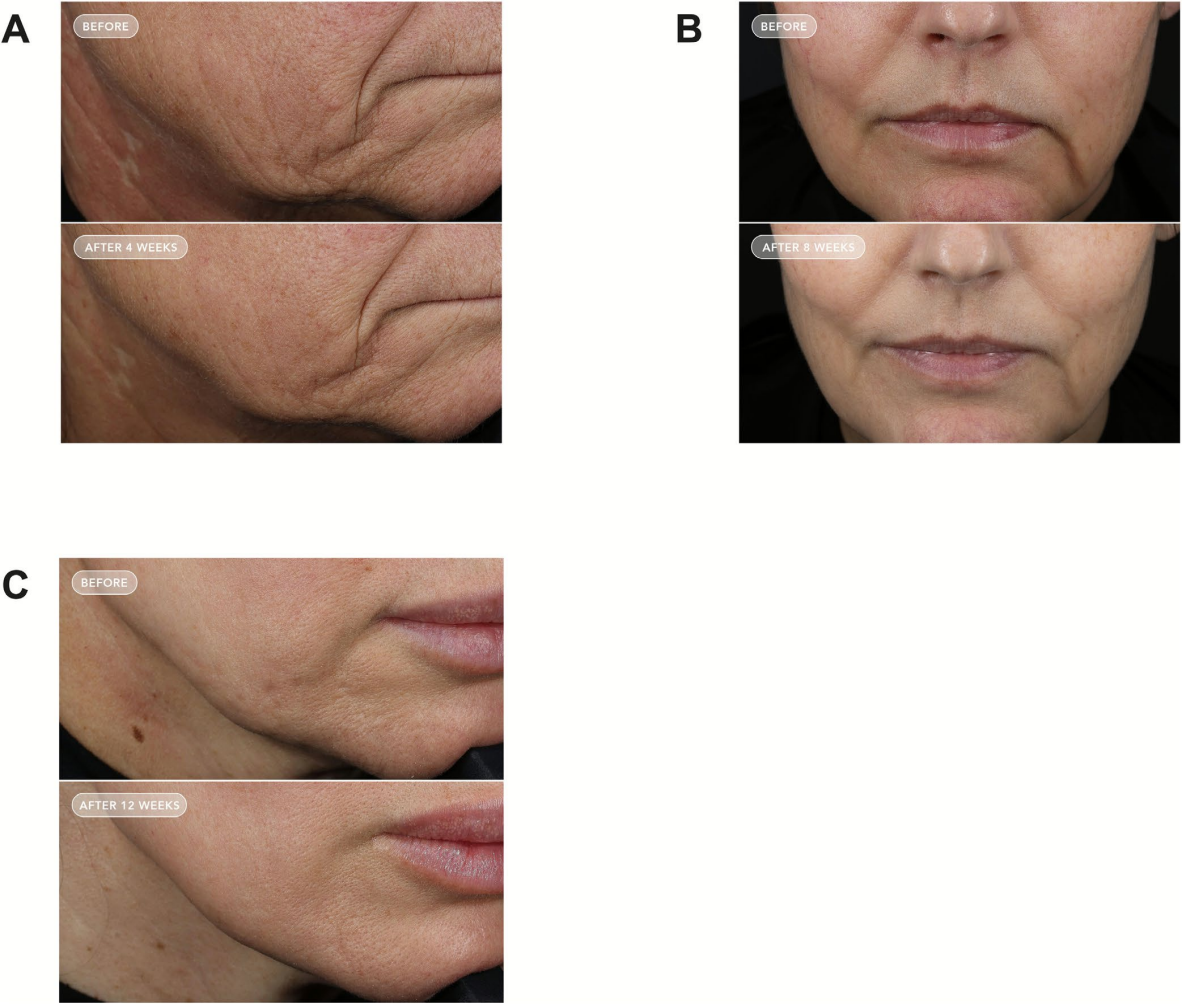
Facial photography with close‐up views of the face. (A) Female, 61 years old, 5 months on a GLP‐1 agonist. Images shown from top to bottom at baseline and week 4. (B) Female, 44 years old, 15 months on a GLP‐1 agonist. Images shown from top to bottom at baseline and week 8. (C) Female, 46 years old, 9 months on a GIP/GLP‐1 co‐agonist. Images shown from top to bottom at baseline and week 12.

Photographs of the participants are shown in Figures 7 and 8.

### 3.10 Adverse Events

There were no treatment‐related adverse effects.

## 4 Discussion

Our results indicate that the application of a topical volumizing cream can augment the appearance of facial volume and reduce the signs of aging in those that have undergone significant weight loss. Of note, most participants in this study were on GLP‐1/GIP agonists (semaglutide or tirzepatide), and our results show that facial volume can be partially improved in the setting of the use of these medications.

The results also suggest that the ingredients did work in a synergistic fashion. There was an increase in overall facial volume, an increase in the elasticity measures, an improvement in the skin barrier (reduced TEWL), improved skin hydration, and the reduction in the visible appearance of wrinkles suggesting that the effects of the topical cream were at different levels of the skin. Apart from visible signs of aging, another side effect of rapid weight loss is skin laxity and loss of elasticity [34]. Our results show that multiple measures of skin elasticity were improved suggesting that skin laxity was addressed in addition to facial volume.

In our cohort, week 8 showed a relative decrease in capacitance‐derived hydration compared with weeks 2 and 4 (Figure 6A) and a less favorable TEWL profile (Figure 6B). This pattern is consistent with the literature demonstrating that skin biophysical properties fluctuate over time in response to environmental and weight‐related factors, likely contributing to the non‐significant trough in viscoelasticity and net elasticity at week 8 (Figure 5B,C). Nam et al. reported significant seasonal variation in both corneometer‐measured hydration and cutometer‐derived elasticity while tracking ambient temperature and humidity in healthy women [35]. A study by Green et al. concluded that environmental exposures based on geographic location can alter TEWL, a key determinant of stratum‐corneum hydration [36]. Values remained above baseline at all visits, and week 12 demonstrated a significant improvement versus baseline. The week 12 rise, despite progression into drier months of the study location, is consistent with a cumulative treatment effect on skin volume and barrier function. The significant improvement at week 12, despite progression into drier months, is consistent with cumulative barrier benefits observed with topical regimens that improve stratum corneum water binding and lipid organization, which are associated with lower TEWL and better Cutometer‐derived elasticity over 4–12 weeks [37, 38, 39]. In addition, extracellular matrix remodeling during active weight loss can transiently blunt mechanical measures mid‐course, with recovery as remodeling progresses [40]. These findings support the interpretation that the week‐8 dip in hydration and elasticity likely reflects physiological variability during ongoing weight change and environmental exposure, rather than a loss of efficacy of the topical volumizing regimen.

The subjects' weight change suggests that the overall weight loss observed was largely driven by a single subgroup, raising important considerations for interpreting the volumizing cream's efficacy (Table 3). Across all 33 participants, mean body weight decreased by ~1.68% (*p* = 0.014) over the 12‐week study. Importantly, stratified analysis revealed that only the GIP/GLP‐1 co‐agonist subgroup (*n* = 9) experienced significant weight loss (~3.87%, *p* = 0.028). In contrast, the other three subgroups—GLP‐1 agonist users (*n* = 15), bariatric surgery patients (*n* = 4), and those using other weight‐loss methods (*n* = 4) – showed no statistically significant weight change over the study period (*p* > 0.05 for each). The overall weight‐loss significance was skewed almost entirely by the GIP/GLP‐1 cohort's reduction, whereas the majority of participants maintained relatively stable weight. This distinction is critical: it indicates that for most subgroups, weight remained essentially constant, isolating the effects of the topical treatment from weight fluctuation confounders. Had the dual‐agonist subgroup not been included, the pooled weight change would not have reached significance, emphasizing how this subgroup's weight trend influenced the aggregate results.

**TABLE 3:** Average percent weight change at week 12 compared to baseline.

Group	Average % weight change	SEM	*p*
GLP‐1 agonist (*n* = 15)	−1.15%	0.84%	0.1522
GIP/GLP‐co‐agonist (*n* = 9)	−3.87%	1.42%	0.0282
Gastrectomy sleeve (bariatric) surgery (*n* = 5)	−1.09%	1.63%	0.5665
Other (4)	0.54%	1.12%	0.5356
Combined (*n* = 33)	−1.68%	0.64%	0.0135

**TABLE 4:** Tolerability was assessed in the form of a questionnaire at week 2, week 4, week 8, and week 12.

	None 0	Mild 1	Moderate 2	Severe 3
Week 2				
Itching	96.97%	0.00%	3.03%	0.00%
Burning	93.94%	6.06%	0.00%	0.00%
Stinging	96.97%	0.00%	3.03%	0.00%
Redness	90.91%	9.09%	0.00%	0.00%
Hypopigmentation	96.97%	3.03%	0.00%	0.00%
Hyperpigmentation	100%	0.00%	0.00%	0.00%
Week 4				
Itching	96.97%	3.03%	0.00%	0.00%
Burning	93.94%	6.06%	0.00%	0.00%
Stinging	96.97%	3.03%	0.00%	0.00%
Redness	85.85%	12.12%	3.03%	0.00%
Hypopigmentation	96.97%	3.03%	0.00%	0.00%
Hyperpigmentation	93.94%	3.03%	3.03%	0.00%
Week 8				
Itching	100%	0.00%	0.00%	0.00%
Burning	100%	0.00%	0.00%	0.00%
Stinging	100%	0.00%	0.00%	0.00%
Redness	90.63%	9.38%	0.00%	0.00%
Hypopigmentation	96.88%	3.13%	0.00%	0.00%
Hyperpigmentation	96.88%	3.13%	0.00%	0.00%
Week 12				
Itching	100%	0.00%	0.00%	0.00%
Burning	100%	0.00%	0.00%	0.00%
Stinging	100%	0.00%	0.00%	0.00%
Redness	93.10%	6.90%	0.00%	0.00%
Hypopigmentation	100%	0.00%	0.00%	0.00%
Hyperpigmentation	100%	0.00%	0.00%	0.00%

The effects of this cream were observed even in the setting of significant weight loss. That is notable as this is a more difficult population to improve signs of facial aging as they are actively taking a medication that would counteract any potential effects of a topical cream. While this cream was studied specifically in those with significant weight loss, the effects would likely not be restricted to those with ongoing weight loss and future studies in those without significant weight loss but with typical age‐related changes in facial aging would be warranted.

This study had several limitations. The study was conducted over a period of 12 weeks, and longer‐term results cannot be deduced from this study. The topical regimen included a sunscreen in addition to the topical volumizing cream. However, sunscreens are not known to affect facial volume to the levels that were noted in this study. This study was designed to assess how facial aging can be shifted after weight loss has already occurred. What is not known is if this cream can be used prophylactically prior to commencement of any weight loss and should be the focus of future studies.

## 5 Conclusion

Our results show that the twice daily use of a volumizing cream (Vol.U.Lift) can improve multiple parameters of facial aging in those that have undergone significant weight loss. The improvements include improvements in the appearance of facial volume and wrinkles as well as skin biophysical features such as skin elasticity, hydration, and barrier.

## Author Contributions

Conception and design: R.K.S., N.A. Data acquisition: N.N., A.A.; or analysis and interpretation of data: N.N., N.A., A.L., W.A., M.N.D., R.K.S. Drafting of manuscript: N.N., N.A., A.L., R.K.S.

## Funding

Funding for this study was provided by Image Skincare.

## Disclosure

R.K.S. serves as a consultant or speaker for Burt's Bees, Sanofi, Bristol Myers Squibb, Arbonne, Trace Minerals, Codex Labs, Pfizer, Phothera, Nutrafol, Lilly, Galderma, Incyte, Novartis, Arcutis, Abbvie, Leo, UCB, Sun, and Regeneron Pharmaceuticals. The authors have no disclosures.

## Ethics Statement

The study was conducted according to the guidelines of the Declaration of Helsinki and approved by the Allendale Institutional Review Board (protocol# IRI24_08_ImageSkin_OTIS) on 14 January 2025.

## Consent

Signed informed consent was obtained from all subjects involved in the study. All participants provided written consent for publication of their photographs.

## Conflicts of Interest

R.K.S. has served as a consultant or speaker for Amgen, Burt's Bees, Sanofi, Bristol Myers Squibb, Arbonne, Trace Minerals, Codex Labs, Pfizer, Phothera, Almirall, Nutraceutical Wellness, Lilly, Galderma, Incyte, Novartis, Arcutis, Janssen, Abbvie, Leo, UCB, Sun and Regeneron Pharmaceuticals. The other authors declare no conflicts of interest.

## Data Availability

The data that support the findings of this study are available from the corresponding author upon reasonable request.
